# Treatment of Rubeola by Inunction

**Published:** 1850-11

**Authors:** John Evans

**Affiliations:** Prof. of Obstetrics, &c., in Rush Medical College, etc., etc.


### ARTICLE VII.

*Treatment of Rubeola by Inunction.*—By JOHN EVANS. M.D., Prof. of Obstetrics, &c., in Rush Medical College, etc., etc.

June 1, 1850, Miss F., aged 15 years, was laboring under the symptoms that characterize a violent attack of Measles. The febrile action was strong---pain in the extremities, loins and head, severe---the eyes were injected, suffused and intolerant of light---distressing nausea was constant, and the characteristic eruption was well marked upon the face, neck and breast.

I gave Dovers powder grs. viij. every six hours with free use of warm teas.

Finding no abatement of the distressing symptoms the next morning, I determined to use inunction, and, as practiced by Dr. Schneeman in Scarlatina, directed the patient to be rubbed with a piece of fat bacon over the entire cutaneous surface.

The relief was marked by the subsidence of all the distressing symptoms in a few hours, and the application was repeated twice the next day. No other treatment was applied except the free use of warm teas. The recovery was more rapid than I had before seen in such cases, and without any disagreeable sequel.

Two other members of the same family were treated by the inunction with the same favorable results.

I have since used the plan of treatment in a number of cases and with uniform and prompt relief.

